# *Candida tropicalis* culture supernatants modulate *Pseudomonas aeruginosa* antimicrobial resistance and biofilm formation

**DOI:** 10.1038/s41598-025-31858-6

**Published:** 2025-12-13

**Authors:** Chandni Sachdeva, Sujan Prakash Acharya, Anand Sairam, Thokur Sreepathy Murali

**Affiliations:** Department of Public Health Genomics, Manipal School of Life Sciences, Manipal Academy of Higher Education, Manipal, Karnataka India

**Keywords:** *Pseudomonas aeruginosa*, *Candida tropicalis*, Biofilm formation, Antimicrobial resistance, Microbial metabolites, Microbial cross-talk, Diseases, Microbiology

## Abstract

Polymicrobial infections involving *Pseudomonas aeruginosa* (PA) and *Candida tropicalis* (CT) majorly contribute to persistent infections resulting in challenges to effectively treat chronic wounds, such as diabetic foot ulcers (DFUs). We investigated the interactions between PA and CT, especially on the role of microbial metabolites in modulating biofilm formation, growth dynamics, antimicrobial susceptibility, and gene expression in co-habitants. Using clinical isolates from DFUs, we examined the effects of microbial supernatants on biofilm formation, microbial growth, and resistance to antibiotics and antifungals. Additionally, we assessed the expression of resistance genes (*aph(3’)-IIb* and *gyrA*) in PA in response to treatment with cell-free CT supernatant. Our findings revealed strain-specific interactions between PA and CT. Supernatants from high biofilm forming CT significantly affected biofilm formation and growth in PA, while PA supernatants universally suppressed CT growth. Notably, low biofilm forming strain of PA exhibited enhanced biofilm formation and growth when treated with supernatant from low biofilm forming CT, suggesting a cooperative interaction. Antimicrobial susceptibility assays demonstrated that CT supernatants modulated resistance to aminoglycosides and fluoroquinolones in PA, with *aph(3’)-IIb* and *gyrA* gene expression being significantly upregulated. Conversely, PA supernatants sensitized CT to antifungals, particularly amphotericin B and fluconazole. The results underscore the importance of understanding interspecific interactions in polymicrobial infections. Our results highlight the complex interplay between PA and CT, driven by microbial metabolites that influence biofilm formation, growth, and antimicrobial resistance and provides fresh insights into the mechanisms underlying PA*-*CT interactions and their implications for chronic wound management.

**Supplementary Information:**

The online version contains supplementary material available at 10.1038/s41598-025-31858-6.

## Introduction

Polymicrobial infections, particularly those involving bacteria and fungi, are increasingly recognized as a significant clinical challenge, especially as seen in chronic wounds, cystic fibrosis, and immunocompromised patients^1^. Among these, co-infections involving *Pseudomonas aeruginosa* and *Candida* species are particularly concerning due to their ability to form high levels of biofilms, resist antimicrobial therapies, and modulate each other’s virulence and survival mechanisms^2,3^. *Pseudomonas aeruginosa*, a Gram-negative opportunistic pathogen, and *Candida tropicalis*, an emerging fungal pathogen, have been frequently isolated from DFUs^4,5^, where they engage in complex interactions that exacerbate disease severity and complicate treatment decisions^6^.

Biofilm formation is a key virulence factor for both PA and CT, providing protection against host immune responses and antimicrobial agents^7,8^. Microbial metabolites play a crucial role in shaping the dynamics of polymicrobial infections, by modulating growth, biofilm formation, and antimicrobial susceptibility, often in a strain-specific manner^9,10^. In polymicrobial biofilms, these species can interact in synergistic or antagonistic manner, influencing each other’s biofilm-forming capacity and overall virulence and fitness. Co-infections involving PA and *Candida* species are often associated with increased resistance to antibiotics and antifungals, partly due to the exchange of resistance genes and production of protective biofilms^1,5,11^. For instance, *Candida albicans* produces farnesol, a quorum-sensing molecule that inhibits PA biofilm formation, while PA produces phenazines that inhibit *C. albicans* growth^12,13^. Similarly, PA can utilize ethanol produced by *C. albicans* as a carbon source, promoting its growth, while clinical isolates of PA have been reported to increase biovolume of *C. albicans* in co-cultures^9,14^. Reports also suggest that PA can enhance the resistance of *C. albicans* to fluconazole via production of small molecules, while *C. albicans* can modulate the expression of antibiotic resistance genes in PA^15,16^. These reports highlight the importance of studying microbial metabolites in the context of polymicrobial infections, as they can significantly influence infection outcomes and treatment efficacy. However, interactions between PA and CT remain poorly understood, despite the clinical relevance of CT in chronic infections.

This study aims to address these gaps by investigating the interactions between PA and CT especially in the context of biofilm formation, growth dynamics, antimicrobial susceptibility (AMS), and gene expression modulation. Using clinical isolates from diabetic foot ulcers, we explore how microbial cell-free supernatants influence these interactions, providing insights into polymicrobial infections.

## Methodology

### Strains and media

ATCC strains of PA (ATCC 27853), CT (ATCC 750) as well as clinical isolates of these species isolated from DFU were included in the study. The DFU isolates were cultured from swab samples collected from consenting diabetic patients who visited Kasturba Hospital, Manipal Academy of Higher Education, Manipal. The study protocol was approved by the Institutional Ethics Committee (IEC/2019/087), Kasturba Hospital, Manipal, registered with the Clinical Trials Registry - India (CTRI/2020/01/022859) and was carried out in accordance with the declaration of Helsinki. Written informed consent was obtained from all the subjects recruited in the study. Two clinical strains each of PA and CT were selected based on levels of biofilm produced in *in vitro* crystal violet assay. Table 1 includes a list of strains used in this study.

**Table 1:** Strains used in this study.

Species	Strain	Source	Remarks
*Pseudomonas aeruginosa*	PA-ATCC	PA ATCC 27853	High biofilm forming
	PA-LBF	DFU	Low biofilm forming
	PA-HBF	DFU	High biofilm forming
*Candida tropicalis*	CT-ATCC	CT ATCC 750	High biofilm forming
	CT-LBF	DFU	Low biofilm forming
	CT-HBF	DFU	High biofilm forming

All strains were grown in Mueller Hinton broth (MHB) and agar (MHA) (HiMedia) for crystal violet biofilm assay as well as for antimicrobial susceptibility (AMS) testing. PA strains were tested against antibiotics Amikacin (AMK), Ciprofloxacin (CIP) and Gentamicin (GEN) while CT stains were tested against antifungals Ketoconazole (KT), Fluconazole (FLC) and Amphotericin-B (AMB).

### Sample preparation

Monocultures were grown in 5 mL of MHB by adding 20 µL of microbial culture (OD_600_ = 0.1) and kept in shaker-incubator at 37 °C for 48 h to target secondary metabolite production. The cells were then quantified using a Neubauer hemocytometer and cell concentrations were adjusted to OD_600_ = 1 by adding sterile medium. From this dilution, equal volumes of cultures were taken for further processing. The cell suspension was mixed thoroughly, and a cell-free supernatant was obtained by centrifugation at 9,600 xg for 5 min, followed by filtration twice with 0.2 micron filter. The supernatant filtrate was subsequently inoculated onto MHA and incubated at 37 °C for 48 h to assess the presence of any microbial growth.

The filtrate was utilized immediately for subsequent experiments. For interaction studies aimed at evaluating the effect of microbial metabolites on the co-inhabitant (e.g., the effect of PA-LBF supernatant on CT-LBF), a mixture was prepared by combining equal volume of fresh media and PA-LBF supernatant. To this mixture, 1/10th of volume of CT-LBF culture was then added, which was earlier adjusted to an OD_600_ of 0.1. The same protocol was followed for microbial growth study, the biofilm formation assay and the AMS testing.

### Microbial growth and absorbance measurements

All strains were grown at 37 °C in a shaking incubator in MHB overnight prior to growth experiments. The following morning, cultures were diluted (OD_600_ = 0.1) and added to 5 mL of MHB and the microbial growth was monitored by measuring the absorbance at 600 nm after 24 h.

### Crystal Violet biofilm assay

The overnight-grown culture was diluted to OD_600_ of 0.01 with fresh medium and 20 µL of this diluted culture was added to individual wells of sterile, polystyrene, 96-well, flat-bottom tissue culture plates containing 90 µL of MHB and 90 µL of the cell-free supernatant. The plates were incubated for 24 h at 37 °C. The wells were washed four times with 0.2 mL sterile distilled water after removing the contents of each well. The biofilms were stained with 0.1% crystal violet and solubilized using 30% glacial acetic acid. Optical density of the stained biofilm was determined with an ELISA autoreader (Infinite M200; Tecan) at a wavelength of 570 nm^17^. The experiments were performed in triplicate. Wells with only the MHB served as negative controls. Wells containing 180 µL of MHB supplemented with 20 µL of the diluted culture (adjusted to OD_600_ = 0.1) served as growth controls. The threshold for high biofilm production was established at OD_570_ = 0.5 in our biofilm assays.

### Microbroth dilution assay

20 µL of PA strains (PA-ATCC, PA-LBF, and PA-HBF), adjusted to an optical density (OD_600_) of 0.1, were inoculated in 80 µL MHB in a 96-well plate at 37 °C for 24 h at 220 rpm. This was supplemented with 80 µL of the respective cell-free supernatants from CT strains (CT-ATCC, CT-LBF, and CT-HBF), along with 20 µL of antibiotic solution (GEN, AMK and CIP). AMS testing was performed using a concentration range of 0.01–2 µg/mL, based on preliminary MIC estimations obtained through HiComb strip assays (HiMedia). This range was selected as it encompassed the MIC values for most strains, while others exhibited resistance according to CLSI interpretive breakpoints. The experimental setup included sterility controls (medium alone), growth controls (culture without antibiotics), and samples with antibiotics but without supernatant addition to ensure comprehensive evaluation.

Similarly, experiments were conducted for CT strains (CT-ATCC, CT-LBF, and CT-HBF) by inoculating them in MHB in a 96-well plate with the respective cell-free supernatants from PA strains (PA-ATCC, PA-LBF, and PA-HBF) and antifungal agents (AMB, FLC and KT). The experiments were performed in triplicates.

### Antimicrobial resistance (AMR) associated gene expression analysis

To analyse the influence of cell-free supernatants on AMR, strains PA-LBF and PA-HBF were treated with CT supernatants (CT-ATCC, CT-LBF, or CT-HBF) and cultured for 24 h at 37 °C and 220 rpm in MHB. Total RNA was extracted using TRIzol reagent (Invitrogen, ThermoFisher Scientific) following the protocol described by Rio et al.^18^. The concentration and purity of the isolated RNA were assessed using spectrophotometric analysis. To eliminate genomic DNA contamination, the RNA samples were treated with DNase I (Ambion, Invitrogen). cDNA synthesis was carried out using the High-Capacity cDNA Reverse Transcription Kit (Applied Biosystems, ThermoFisher) under the following conditions: incubation at 25 °C for 10 min, 37 °C for 2 h, and 85 °C for 5 s.

The expression levels of AMR genes, *gyrA* and *aph(3’)-IIb* (Table 2), were quantified using quantitative RT-PCR using SYBR Green. The housekeeping gene *rpoD* was used as an internal reference to normalize gene expression data.

**Table 2:** Primers used in expression analysis of antimicrobial resistance associated genes.

Gene	Sequence
*gyrA*	F; TCACCGACGAAGAGTTGATG
	R; CTCTTCGATCTCGGTCTTGG
*aph(3’)-IIb*	F; ATGCATGATGCAGCCACCTCCAT
	R; CACTTCCTGCTTGACGAACAG
*rpoD*	F; GGGCGAAGAAGGAAATGGTC
	R; CAGGTGGCGTAGGTGGAGAA

### Statistical analysis

The quantitative variables were expressed as mean ± standard deviation (SD). The statistical analyses including Student’s t-test and one-way ANOVA with Welch’s correction were performed using GraphPad Prism (version 9.0).

## Results

### Effect of cell-free supernatants on microbial growth

**Fig. 1:**
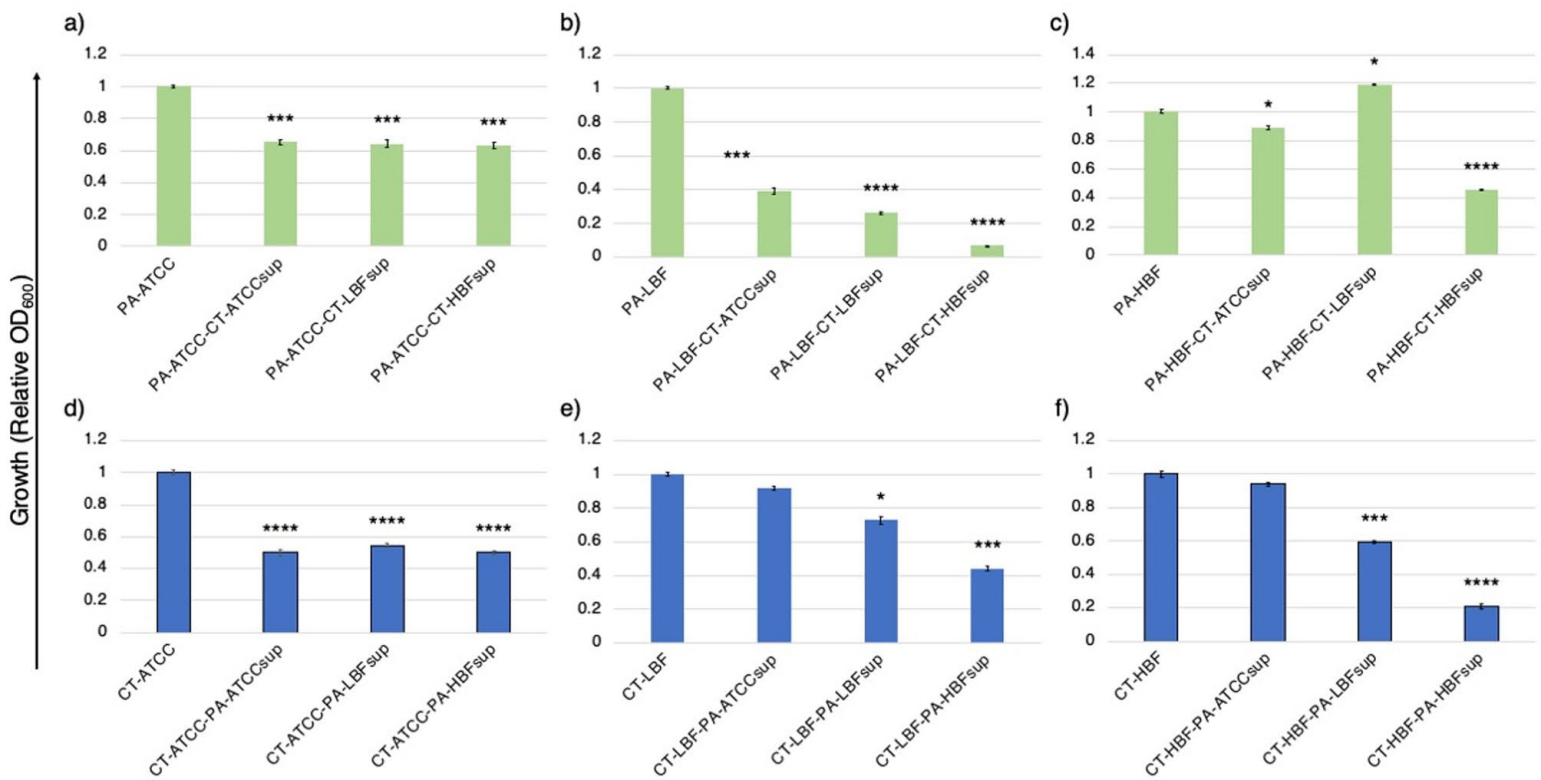
Effect of microbial supernatants on growth (OD₆₀₀) of co-cultured strains. Bar graphs show relative growth of PA and CT strains after treatment with cell-free supernatants (OD_600_) ± SD for PA strains (green bars) **(a)** PA-ATCC, **(b)** PA-LBF, **(c)** PA-HBF cultured with CT supernatants and CT strains (blue bars) **(d)** CT-ATCC, **(e)** CT-LBF, **(f)** CT-HBF cultured with PA supernatants. Data represent mean values from triplicates, with error bars indicating SD. Significant differences (*p < 0.05, **p < 0.01, ***p < 0.001, ****p < 0.0001) compared to respective monoculture controls were determined by one-way ANOVA with Welch’s correction.

The growth of PA strains varied significantly (*p* < 0.05) when exposed to CT cell-free supernatants (sup) in MHB (Supplementary data). The relative growth as measured by absorbance at 600 nm were highest for monoculture PA-ATCC, followed by PA-HBF and PA-LBF. Exposure to CT-ATCCsup generally suppressed growth across all strains (Fig. 1). PA-ATCC growth was reduced by 16% when treated with CT-ATCCsup, by 16% with CT-LBFsup, and by 40% in the presence of CT-HBFsup. PA-LBF exhibited the most pronounced inhibition, with growth declining by 29% in the presence of CT-ATCCsup, 53% with CT-LBFsup, and 71% with CT-HBFsup. PA-HBF growth was less affected, showing a 10% reduction in the presence of CT-ATCCsup but showed a 51% increase in the presence of CT-LBFsup (1.631 ± 0.017), suggesting a strain-specific stimulatory effect. However, PA-HBF growth was suppressed by 54% in the presence of CT-HBFsup when compared to grown alone.

Monoculture growth for CT strains were highest for CT-ATCC and CT-HBF, while CT-LBF grew comparatively less. Treatment with PA supernatants inhibited growth in all the strains tested, though the extent varied (Fig. 1). CT-ATCC growth was reduced by 46% when grown with PA-ATCCsup, by 42% in the presence of PA-LBFsup, and by 60% by PA-HBFsup. CT-LBF growth was suppressed by 15% in the presence of PA-ATCCsup, by 33% with PA-LBFsup, and by 51% when growth with PA-HBFsup. CT-HBF exhibited the most striking inhibition, with growth declining by 13% in the presence of PA-ATCCsup, by 32% in the presence of PA-LBFsup, and by 85% in the presence of PA-HBFsup (Fig. 1).

### Effect of cell-free supernatant on biofilm formation

**Fig. 2:**
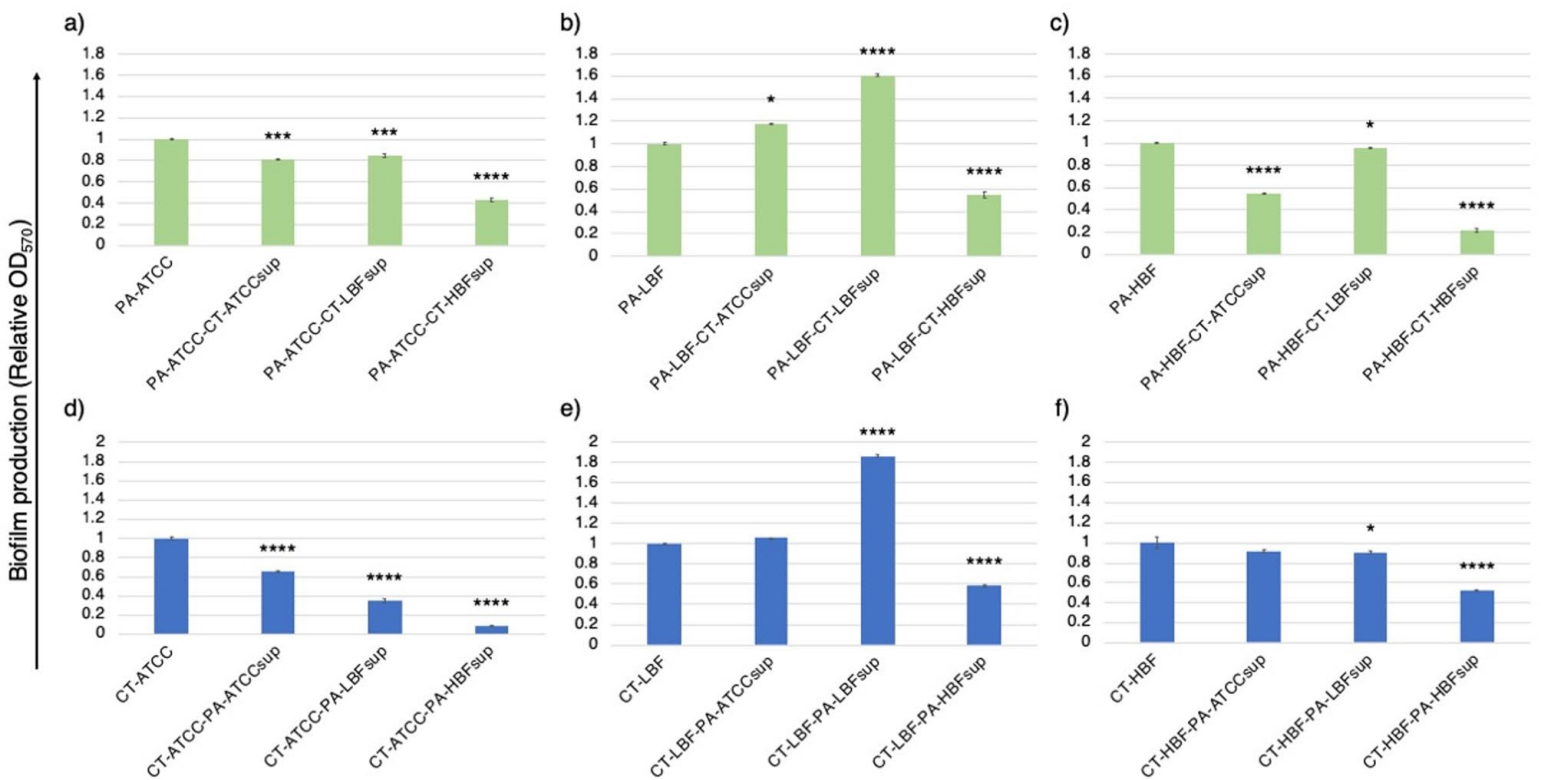
Biofilm formation of PA and CT strains in response to microbial supernatants. Bar graph showing the relative mean optical density (OD_570_) ± SD of crystal violet-stained biofilms for PA strains (green bars) **(a)** PA-ATCC, **(b)** PA-LBF, **(c)** PA-HBF cultured with CT supernatants and CT strains (blue bars) **(d)** CT-ATCC, **(e)** CT-LBF, **(f)** CT-HBF cultured with PA supernatants. Data represent mean values from triplicates, with error bars indicating SD. Significant differences (*p < 0.05, **p < 0.01, ***p < 0.001, ****p < 0.0001) compared to respective monoculture controls were determined by one-way ANOVA with Welch’s correction.

The three PA strains (PA-ATCC, PA-LBF, PA-HBF) showed different levels of biofilm formation in monocultures, with PA-ATCC and PA-HBF exhibiting high biofilm-forming capacity (OD_570_ > 0.5), while PA-LBF produced relatively lower levels of biofilm. Growth in the presence of CT supernatants elicited strain-specific responses. Biofilm formation in PA-ATCC decreased significantly (*p* < 0.001) in the presence of CT-ATCCsup (19%), CT-LBFsup (16%), and CT-HBFsup (57%). In contrast, PA-LBF biofilm increased significantly by 18% in the presence of CT-ATCCsup (*p* < 0.05) and by 61% in the presence of CT-LBFsup (*p* < 0.0001). PA-HBF biofilm was reduced by 5% when grown with CT-LBFsup (*p* < 0.05) compared to the monoculture, while it was significantly suppressed (*p* < 0.0001) in the presence of CT-ATCCsup (45%) and CT-HBFsup (78%). Notably, CT-HBFsup significantly inhibited biofilm formation (*p* < 0.0001) across all PA strains (Fig. 2).

Biofilms produced by CT-ATCC and CT-HBF monocultures were comparatively higher than CT-LBF (Fig. 2). In case of CT-ATCC, biofilm production was significantly (*p* < 0.0001) suppressed by PA-ATCCsup (35%), PA-LBFsup (65%), and PA-HBFsup (91%). CT-LBF biofilm was enhanced by PA-ATCCsup (6% increase) and PA-LBFsup (86% increase; *p* < 0.0001) but was reduced when treated with PA-HBFsup (42%; *p* < 0.0001). For CT-HBF, while all PA supernatants reduced biofilm levels compared to monoculture, a significant reduction (*p* < 0.0001) was observed when treated with PA-HBFsup (47% reduction).

### Effect of cell-free supernatant on antimicrobial susceptibility

**Fig. 3:**
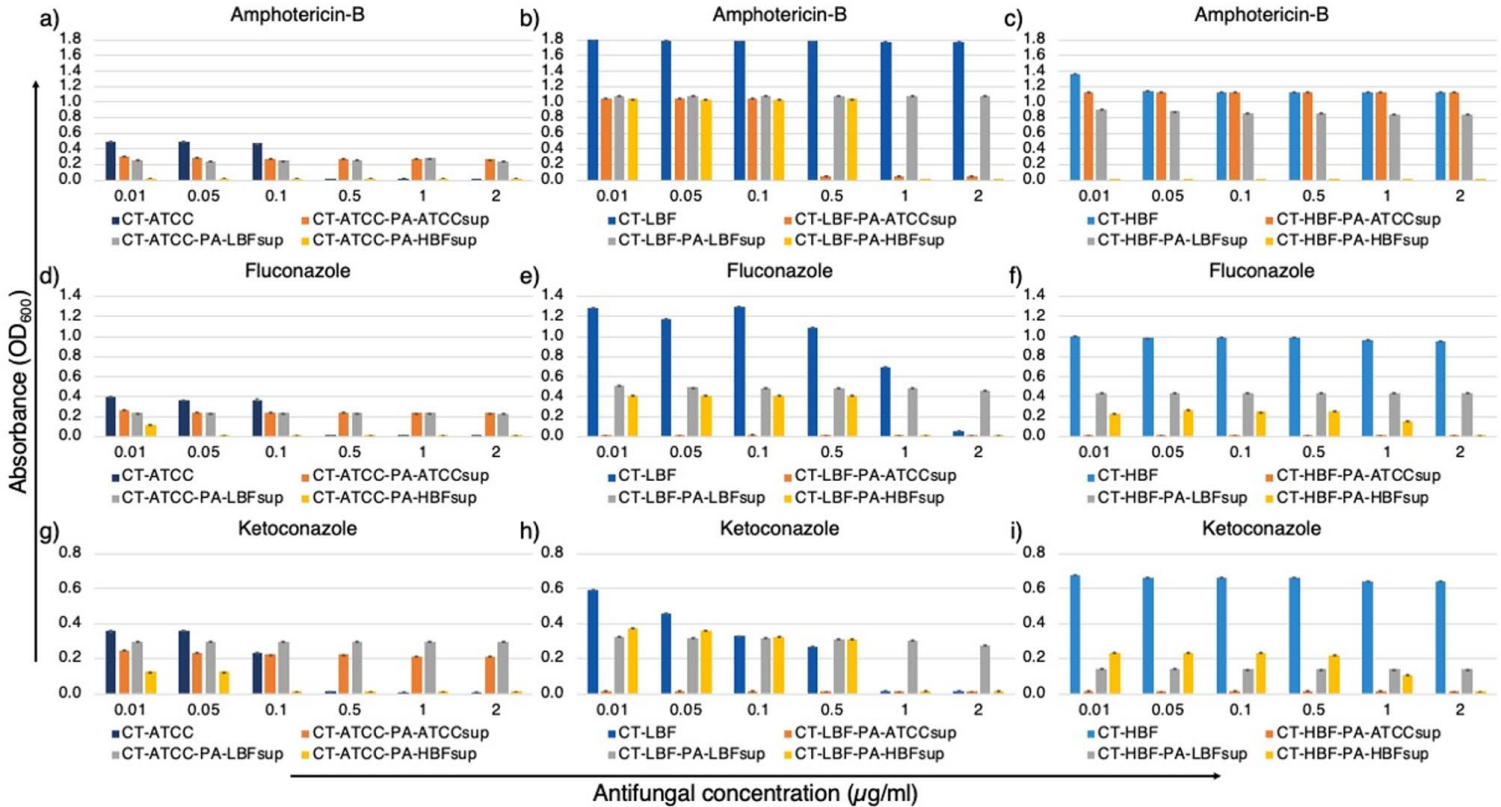
Antimicrobial susceptibility patterns of CT strains to antifungal agents (µg/mL) in the presence of PA supernatants. Bar graphs showing mean growth (OD_600_) ± SD of CT strains- CT-ATCC in PA supernatants with **(a)** AMB **(b)** FLC **(c)** KT, CT-LBF in PA supernatants with **(d)** AMB **(e)** FLC **(f)** KT and CT-HBF in PA supernatants with (g) AMB (h) FLC (i) KT. All data represent mean values from triplicate experiments with error bars indicating SD. Asterisks denote statistically significant differences (*p < 0.05, **p < 0.01, ***p < 0.001, ****p < 0.0001) compared to treatment without bacterial supernatants (one-way ANOVA with Welch’s correction).

The susceptibility of CT strains to AMB concentrations (0.5–2 µg/mL) varied depending on exposure to PA supernatants. Treatment with PA-HBFsup rendered all three CT strains (especially CT-HBF), highly susceptible to AMB. Similarly, treatment with PA-HBFsup rendered CT-ATCC highly susceptible to FLC, while treatment with PA-ATCCsup and PA-HBFsup made CT-LBF highly susceptible to the antifungal compound. In case of ketoconazole, treatment with PA-HBFsup rendered CT-ATCC highly susceptible, while for CT-LBF, treatment with PA-ATCCsup and PA-HBFsup significantly increased its susceptibility (Fig. 3).

**Fig. 4:**
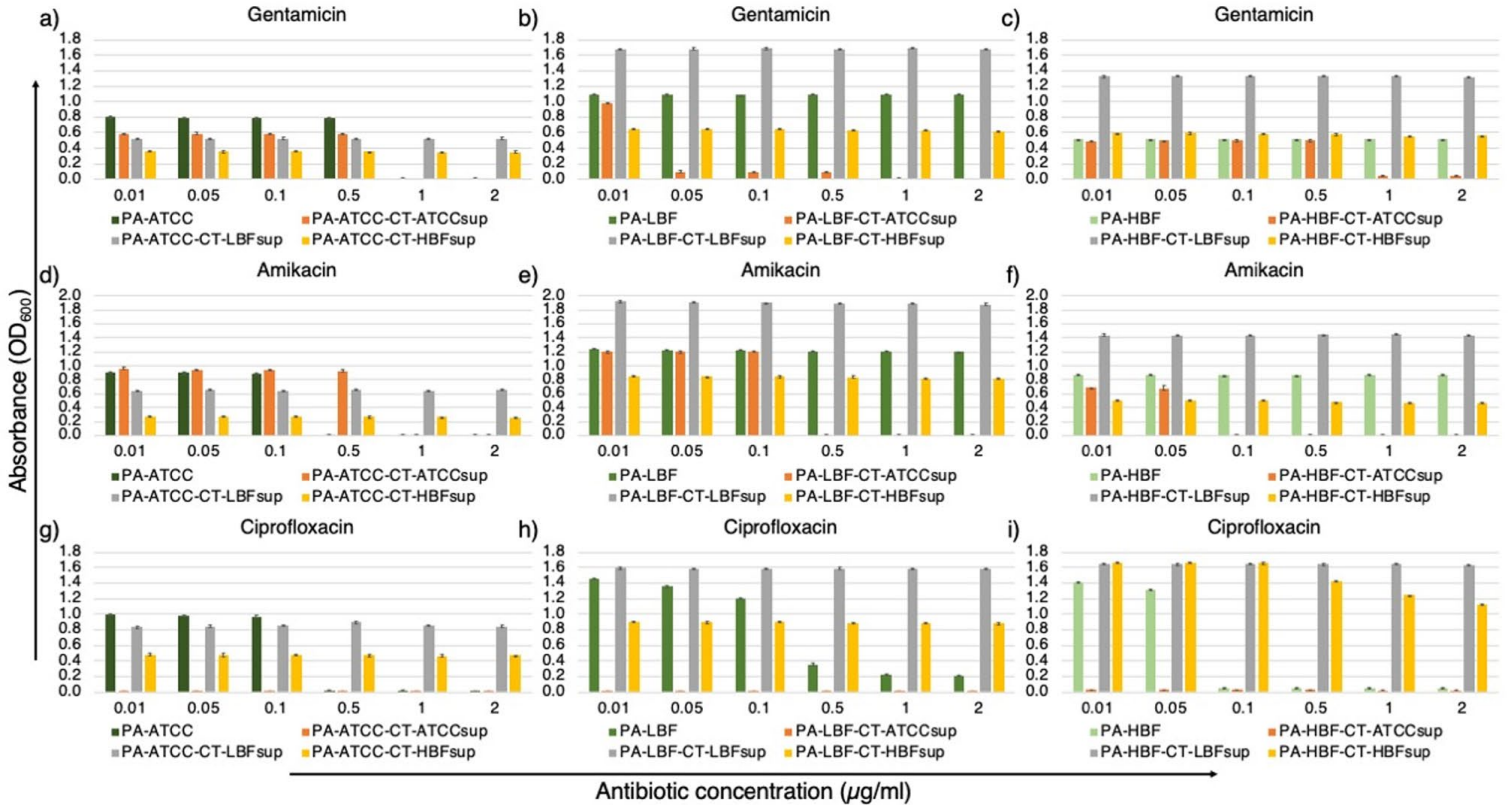
Antimicrobial susceptibility patterns of PA strains to antimicrobial agents (µg/mL) in presence of CT supernatants. Bar graphs showing mean growth (OD_600_) ± SD of PA strains- PA-ATCC in CT supernatants with **(a)** GEN **(b)** AMK **(c)** CIP, PA-LBF in CT supernatants with **(d)** GEN **(e)** AMK **(f)** CIP and PA-HBF in CT supernatants with **(g)** GEN **(h)** AMK **(i)** CIP. All data represent mean values from triplicates with error bars indicating SD. Asterisks denote statistically significant differences (*p < 0.05, **p < 0.01, ***p < 0.001, ****p < 0.0001) compared to respective antifungal-treated controls without bacterial supernatants (one-way ANOVA with Welch’s correction).

The susceptibility of PA strains to gentamicin varied with PA-ATCC exhibiting MIC of 1 µg/mL, when grown alone. However, when treated with CT-LBFsup or CT-HBFsup it showed resistance to the antibiotics at all concentrations. PA-LBF showed high resistance to GEN in monoculture across all concentrations but its growth in the presence of CT-ATCCsup or CT-HBFsup increased its susceptibility. However, treatment with CT-LBFsup enhanced resistance of PA-LBF to GEN at all concentrations. Similarly, treatment with CT-LBFsup significantly increased resistance of PA-HBF to GEN. In case of amikacin, PA-ATCC monoculture showed MIC of 0.5 µg/mL, but CT-HBFsup treatment increased its susceptibility. PA-LBF and PA-HBF both exhibited high resistance to AMK in monoculture, but treatment with CT-ATCCsup made PA-LBF and PA-HBF completely sensitive to AMK. Treatment of PA-LBF and PA-HBF with CT-HBFsup slightly increased its susceptibility to AMK while, when grown with CT-LBFsup increased the resistance of both strains to AMK. In case of CIP, all PA strains show increased susceptibility towards the antibiotic when grown with CT-ATCCsup and showed resistance to the antibiotic when treated with CT-LBFsup and CT-HBFsup at all concentrations (Fig. 4).

### Expression of AMR genes in PA strains

**Fig. 5:**
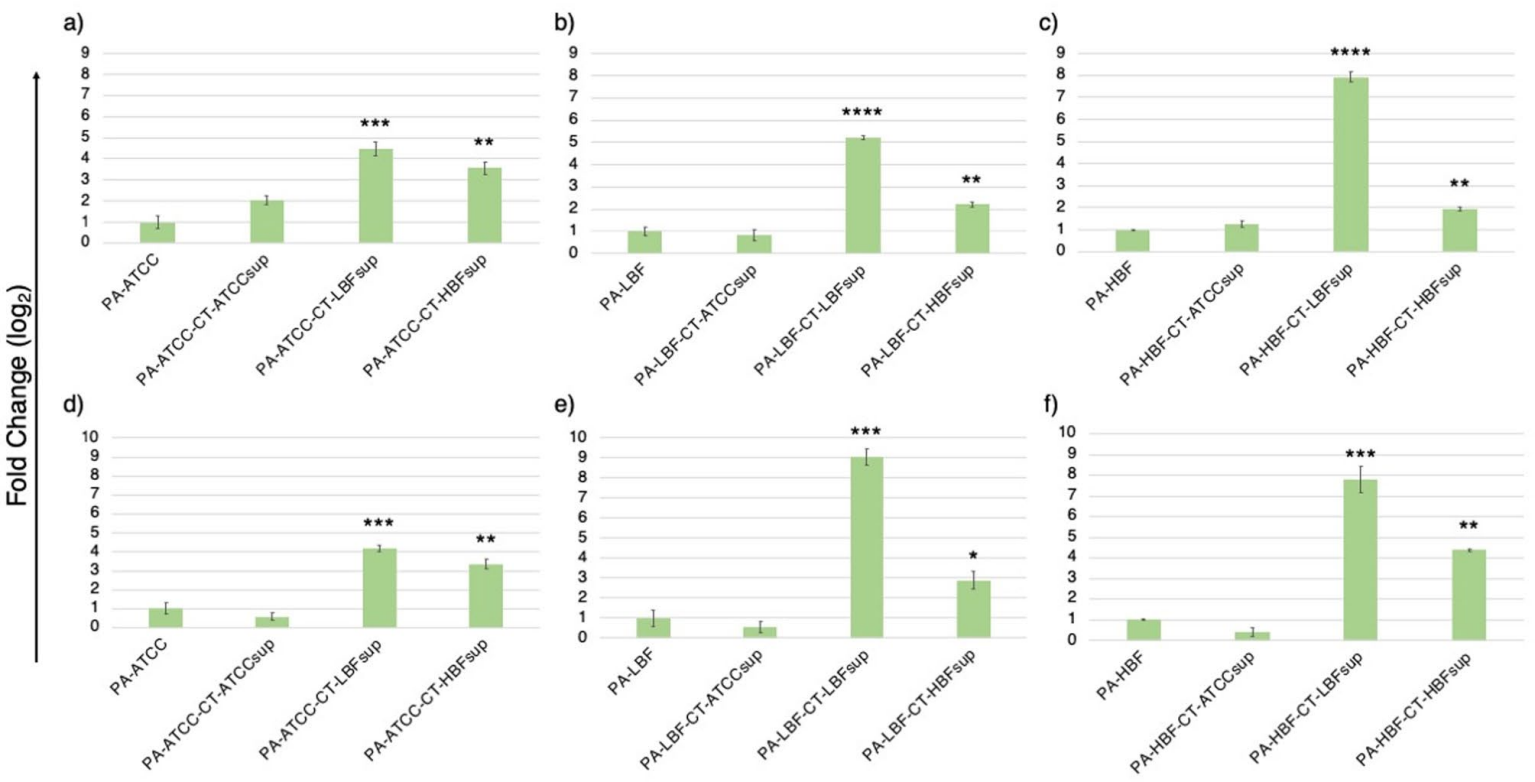
Expression of antimicrobial resistance genes in PA strains in response to CT supernatants. Bar graphs showing relative fold-change in expression (mean ± SD) of *aph(3’)-IIb* in **(a)** PA-ATCC **(b)** PA-LBF **(c)** PA-HBF and *gyrA* in **(d)** PA-ATCC **(e)** PA-LBF **(f)** PA-HBF when treated with CT supernatants. All data was generated from mean values from duplicate experiments with error bars indicating standard deviation. Gene expression was normalized to the housekeeping gene *rpoD* and calculated using 2^(-ΔΔCt) method. Baseline expression was normalized to 1 for monoculture control group. Asterisks denote statistically significant differences (*p < 0.05, **p < 0.01, ***p < 0.001, ****p < 0.0001) compared to respective untreated controls (one-way ANOVA with Welch’s correction).

The expression of *aph(3’)-IIb* gene associated with aminoglycoside resistance^19,20^ was significantly modulated in PA strains when exposed to CT supernatants (Fig. 5a-c). In case of PA-ATCC, treatment with either CT-LBF or CT-HBF supernatants upregulated *aph(3’)-IIb* expression significantly (*p* < 0.01), while for PA-LBF, treatment with CT-LBF and CT-HBF resulted in increased expression with fold changes of 5.21 (*p* < 0.0001) and 2.22 (*p* < 0.01), respectively. For PA-HBF, treatment with CT-ATCCsup resulted in slight upregulation of expression (1.25-fold; ns) and significant increase in gene expression on treatment with CT-LBFsup and CT-HBFsup (7.91-fold; *p* < 0.0001 and 1.94-fold; *p* < 0.01, respectively). However, for PA-LBF, treatment with CT-ATCCsup led to reduced expression of *aph(3’)-IIb* (0.83 fold).

The expression of *gyrA* gene, associated with fluoroquinolone resistance^21,22^, was also significantly altered in response to treatment with CT supernatants (Fig. 5d-f). All PA strains exhibited significant upregulation (*p* < 0.05) in expression of *gyrA* gene, when grown in the presence of CT-LBFsup and CT-HBFsup. In case of PA-LBF, treatment with CT-LBFsup and CT-HBFsup resulted in increased expression with fold changes of 9.07 (*p* < 0.001) and 2.90 (*p* < 0.05), respectively, while for PA-HBF, the treatment with these two supernatants resulted in fold changes of 7.79 (*p* < 0.001) and 4.38 (*p* < 0.01) and respectively.

## Discussion

Co-infections involving PA and CT are increasingly recognized in clinical settings, particularly in chronic wounds such as DFUs. These polymicrobial infections are often more challenging to treat due to complex interactions between the co-existing species, that result in metabolic cross-talk, altered biofilm formation, and modulation of AMR^23^. Our study provides insights into the interactions between PA and CT, focusing on biofilm formation, growth dynamics, antimicrobial susceptibility, and the expression of resistance genes in response to microbial metabolites.

One of the key findings of this study is how biofilm formation can be modulated by pathogens in response to microbial supernatants in a strain-specific manner. For instance, PA-ATCC, PA-LBF and PA-HBF strains exhibited high biofilm-forming capacity in monoculture, which was modulated by CT supernatants. Notably, cell-free supernatant from CT-HBF, a high biofilm producer and multidrug resistant strain, reduced biofilm formation across all PA strains (Fig. 2), suggesting that CT-HBF may produce metabolites that interfere with PA biofilm development resulting in an antagonistic interaction. This observation aligns with previous studies demonstrating that *Candida* species can produce farnesol and tyrosol, quorum-sensing molecules that inhibits PA biofilm formation^12,24^. Similarly, PA-HBFsup significantly suppressed biofilm formation in all CT strains studied, indicating a reciprocal inhibitory interaction (Fig. 2). These findings suggest that biofilm modulation may serve as a competitive strategy, especially in specific niches such as a wound milieu.

The microbial growth data further highlight the competitive dynamics between these species. CT supernatants, particularly CT-HBFsup, significantly inhibited the growth of all PA strains (*p* < 0.0001), while PA supernatants suppressed CT growth (Fig. 1). This reciprocal inhibition suggests that both species produce metabolites that limit the proliferation of the other, potentially to compete with other microbes in a highly competitive polymicrobial environment.

Our results corroborate with previous studies showing that microbial interactions can modulate AMR through gene regulation and metabolic adaptation^1^. The antimicrobial susceptibility assays revealed that microbial supernatants significantly altered the resistance profiles of both species. PA-HBFsup universally enhanced susceptibility to antifungals (Fig. 3), suggesting that PA-HBF produces metabolites that sensitize CT to antifungal agents. This finding is particularly relevant given the rising incidence of antifungal resistance in *Candida* species^5^.

Interestingly, low biofilm forming strains like CT-LBF, could modulate the expression of antibiotic resistance genes (*aph(3’)-IIb* and *gyrA*) in PA, potentially contributing to enhanced resistance to aminoglycosides and fluoroquinolones (Fig. 5), while, both PA-LBF-CT-LBFsup and CT-LBF-PA-LBFsup combinations produced higher biofilms (Fig. 2). This behavior suggests that low biofilm formation or reduced virulence factors may drive strains to cooperate for survival. A similar phenomenon was reported by Bergeron et al.^25^, where a quorum-sensing-deficient PA *ΔlasR* mutant was found to enhance *C. albicans* pathogenicity in co-infection. Additionally, PA-HBF growth was uniquely enhanced by CT-LBFsup (Fig. 1), indicating cooperative interaction. Such interactions may be mediated by metabolic cross-feeding, where one species utilizes metabolites produced by the other, as previously observed in PA*-C. albicans* co-cultures^2^.

The RT-PCR data provide mechanistic insights into the observed changes in antimicrobial susceptibility. Our findings suggest that CT supernatants, particularly CT-LBFsup, enhance the expression of *aph(3’)-IIb* and *gyrA* genes, potentially contributing to increased aminoglycoside and fluoroquinolone resistance respectively. The upregulation of *aph(3’)-IIb* and *gyrA* genes in clinical strains of PA exposed to CT supernatants suggests that microbial metabolites can induce resistance mechanisms at the genetic level. This is particularly concerning in the context of chronic infections, where prolonged exposure to microbial metabolites may drive the evolution of resistance. Further experiments with deletion mutant of related antibiotic resistance genes can provide further credence to this finding.

Our findings also underscore the need for strain-level characterization in clinical settings, as strain-specific interactions in polymicrobial infections may influence infection outcomes and treatment efficacy. However, our study has certain limitations that warrant consideration. Strain-to-strain variability in microbial behavior and metabolite production may influence these interactions, and broader validation across diverse clinical isolates is needed to generalize these findings. First, the investigation was conducted using only two clinical strains of each species, which restricts the generalizability of our findings. A larger sample size encompassing a broader range of clinical isolates would be necessary to confirm whether the observed behaviors are indeed strain-specific. Second, the use of MHB as the growth medium may not fully replicate the physiological conditions encountered *in vivo*, potentially influencing the outcomes of microbial interactions. The supernatants utilized in this study were derived from monocultures, which may not fully replicate the metabolite profiles produced during polymicrobial interactions. In the presence of other pathogens, microbial metabolism can be significantly altered, leading to the production of distinct exometabolome that differ from those generated in monoculture (Sachdeva et al., unpublished data). This limitation means that the observed effects of supernatants on biofilm formation, growth, and antimicrobial resistance may not fully reflect the complex metabolic interactions occurring in co-culture or polymicrobial infection scenarios. Further, we did not study the specific metabolites that could modulate the expression of genes conferring antimicrobial resistance or virulence, hence broader metabolic profiling is needed to establish cause-effect relationships. Future studies using targeted metabolomics approaches would probably help us to identify candidate metabolites in *P. aeruginosa* and *C. tropicalis* supernatants that could have contributed to the observed results. Furthermore, the study utilized cell-free supernatants to assess microbial interactions, which may contain residual nutrients or enzymes alongside bioactive metabolites. While we cannot rule out their potential contribution to the observed effects, our experimental design focused on characterizing net phenotypic changes under clinically relevant conditions (i.e., exposure to secreted microbial products). Incorporating appropriate controls (such as heat-inactivated or enzyme-depleted supernatants) can help us in studying specific role of metabolites and their effects on other pathogens. Finally, the *in vitro* nature of this study does not account for host factors, such as immune responses or tissue-specific microenvironments, which could significantly alter the dynamics of PA and CT interactions in *in vivo* conditions. Future studies incorporating *in vivo* models and diverse growth conditions are essential to validate and extend these findings.

In conclusion, this study demonstrates that PA and CT engage in complex interactions mediated by microbial metabolites, which modulate biofilm formation, growth dynamics, and antimicrobial resistance. These interactions are strain-specific and context-dependent, with implications for both competition and cooperation. The findings suggest that high biofilm-forming strains may exert virulence towards other strains, while certain metabolites may sensitize pathogens to antimicrobial agents. Additionally, low biofilm forming or less virulent strains may seek cooperation instead of competition. These insights have significant clinical relevance, particularly for the management of chronic polymicrobial infections such as DFUs. Future studies should explore the specific metabolites involved in these interactions and their potential as therapeutic targets. Additionally, further studies are warranted using *in vivo* models to validate these findings and assess their relevance in clinical settings. These findings could contribute to a deeper understanding of the mechanisms governing their coexistence and competition, which have significant implications for clinical management and therapeutic strategies.

## Supplementary Information

Below is the link to the electronic supplementary material.

Supplementary Material 1

Supplementary Material 2

## Data Availability

16 S rDNA sequences and ITS sequence of the strains used in the study are deposited in NCBI GenBank database under accession numbers PQ732975 (16 S rDNA) and PQ732978 (ITS).
